# Accelerated Hydrothermal Aging and Degradation Mechanism of PE100 Butt-Fusion Welded Joint

**DOI:** 10.3390/ma17225505

**Published:** 2024-11-12

**Authors:** Yingchun Chen, Yanfeng Li, Jie Yang, Yan Xi

**Affiliations:** College of Architecture and Civil Engineering, Beijing University of Technology, Beijing 100124, China; ychen08089@163.com (Y.C.); liyanfeng8541@163.com (Y.L.); yj1998040221@163.com (J.Y.)

**Keywords:** high-density polyethylene, hydrothermal aging, butt-fusion welded joint, temperature

## Abstract

High-density polyethylene (HDPE) pipelines are extensively utilized in energy transportation in the ocean. However, long-term exposure to water can alter the performance of HDPE, potentially leading to pipeline accidents. This study focuses on simulating the aging characteristics of PE100 polyethylene pipeline butt-fusion welded joints (B-FWJs) in water using hydrothermal accelerated aging experiments at various temperature gradients. The performance of the B-FWJ after hydrothermal aging was characterized using scanning electron microscopy (SEM), oxidation induction time (OIT), attenuated total reflectance Fourier transform infrared (ATR FT-IR) spectroscopy, and mechanical testing. Furthermore, this study analyzed the performance characteristics and changes in the micro-molecular chains of an HDPE B-FWJ pipeline following hydrothermal aging. An investigation was conducted into the effects of hydrothermal aging temperature and duration on the physical and chemical characteristics of HDPE B-FWJ, and the aging mechanism under hydrothermal aging conditions was explored. The results indicate that increasing hydrothermal aging temperature leads to a more significant decrease in the mechanical properties of the B-FWJ. These findings contribute to understanding the aging behavior of PE100 pipelines in the joint section and offer insights to mitigate the risks associated with the aging of and damage to B-FWJ pipelines in the ocean.

## 1. Introduction

As one of the most important plastic pipes, high-density polyethylene (HDPE) pipes have well-known advantages such as excellent safety, practicality, durability, and reasonable price [1,2,3]. They have a wide range of applications and uses [4,5]. For example, HDPE is used for energy transportation and raw material transportation in the ocean. At the same time, HDPE pipelines are gradually replacing various metal pipelines in lifeline engineering [6,7,8]. However, irrespective of their application in the ocean, HDPE B-FWJs are exposed to diverse and challenging working environments. Various geological disasters, external environmental interferences, temperature fluctuations due to seasonal changes, and numerous other factors can be encountered. In the event of any accidents, particularly in areas with a concentrated fishing industry or that are near the coast, the consequences can be severe. Due to the secondary processing of the pipe joint position, the performance of HDPE pipes may change, and the various properties at the pipe joint belong to the weak position of the pipe body during the aging process [9,10]. The B-FW method is extensively employed in on-site scenarios due to its fast and convenient application, offering high-quality and reliable pipeline joints [11,12]. There are two aging methods for pipeline joints: natural aging and accelerated aging [13]. However, accelerated aging methods are commonly used to study the degree of polyethylene aging to shorten the experimental cycle and achieve the goal of rapid aging of polyethylene samples in a short period [14]. The mechanical properties of HDPE undergo a decline, and its safe service time is subject to change in actual engineering due to the distinctive molecular structure of the material and the influence of various factors [15]. Therefore, the focus of researchers has become the alterations in performance and reliable operational duration of HDPE pipelines during their operation [16,17].

Currently, the primary methodologies for connecting polyethylene (PE) pipelines are butt-fusion connection (BF) [18] and electric-fusion connection (EF) [19,20]. Both BF and EF joints are created through docking machinery and procedures that involve butt-fusion welding [21]. Consequently, the pipe joint section, being a secondary processing product, exhibits distinct performance characteristics and microstructures compared with the pipe body. In engineering practice, joints are often regarded as the Achilles’ heel of pipeline systems due to their susceptibility to failures [22]. Therefore, the performance of joints is paramount to the overall safety and efficiency of the pipeline system. To this end, researchers have been actively exploring methods to evaluate and enhance the performance of PE joints. Wermelinger and his team designed a water axial tension (HAT) test to assess the performance of PE BF joints with various internal defects. This test provides valuable insights into the strength and durability of BF joints under different conditions [23]. Similarly, Kim and his colleagues conducted notch tensile testing to compare the toughness of B-FWJ with the tube body. Their findings revealed that the toughness of welded joints was inferior to that of the tube body, highlighting the need for further research to improve the welding process and enhance joint performance [24]. Ghanadi et al. conducted a study to investigate the effects of ultraviolet radiation on the degradation of HDPE pile sleeves in both natural and laboratory environments, with the aim of isolating the effects of UV radiation. Compared with samples exposed to accelerated UVB irradiation in the laboratory, those exposed to the marine environment exhibited an increase in oxygen-containing surface functional groups and more pronounced morphological changes [25]. Liu et al. explored the characteristics of polyethylene changes under high-temperature aquatic conditions and found that depolymerization typically occurs subsequent to thermal cracking. During this process, the scission of PE molecules is random and primarily induced by thermal treatment, while reactions such as polymerization, cyclization, and radical recombination based on radical mechanisms are relatively less prevalent [26]. Despite these advancements, there is still a paucity of research on the aging performance of BF joints in marine environments. Given the harsh conditions that marine pipelines endure, including exposure to high temperatures, pressure, and corrosive elements, it is imperative to conduct comparative experimental studies on the effects of hydrothermal aging at different temperatures on the performance of BF joints. Such studies will not only contribute to a better understanding of the aging mechanisms of PE materials in marine environments but also aid in the development of more durable and reliable pipeline systems.

HDPE B-FWJs were taken in this article as the subject of study, and accelerated aging tests were conducted on HDPE B-FWJs under hydrothermal aging conditions at different temperatures. Mechanical testing was conducted on the B-FWJs, the test results were analyzed, and the aging laws of the B-FWJs under different temperature hydrothermal aging conditions were summarized. The analysis focused on the cross-linking mechanism of molecular chains in the region influenced by the heat of HDPE B-FWJs following hydrothermal aging at various temperatures. The findings of this study contribute to addressing the knowledge gap in the aging theoretical system of HDPE pipes, specifically about the B-FWJ under hydrothermal aging conditions. The findings of this paper will contribute to a deeper understanding of the B-FWJ in marine environments.

## 2. Experimental Setup

### 2.1. Material

**Figure 1:**
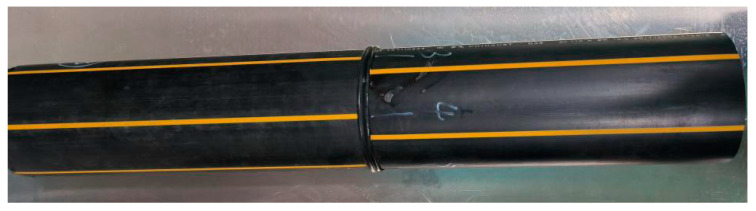
Experimental raw material.

Figure 1 shows the material used in the experiment. It was a PE100 pipeline B-FWJ joint produced by Yada Plastic Products Co., Ltd. (Zhuozhou, China), using HE3490-LS (bimodal polyethylene produced by the Nordic Chemical Industry (Oslo, Norway)) as the raw material, with a nominal diameter (ND) of 110 mm and a standard dimension ratio (SDR) of 11.

### 2.2. Hydrothermal Accelerated Aging Test

The PE100 B-FWJ samples were immersed in three hydrothermal aging chambers, with the temperature set at 40 °C, 60 °C, and 80 °C for each respective chamber, at an aging time of 120–600 h, with samples being taken every 120 h. The samples were taken out of the hydrothermal aging chamber in batches and placed on experimental plates, and allowed to cool to room temperature (23 ± 0.5 °C) for 2 h. After using lint-free paper to absorb surface moisture, the samples were placed in open sample bags and air-dried for 5 days before performance and characterization testing. The accelerated aging plan is shown in Table 1.

### 2.3. Mechanical Performance Testing

**Figure 2:**
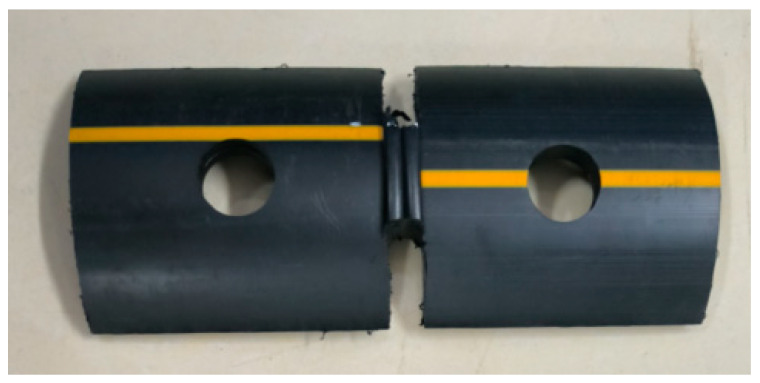
Dumbbell sample for tensile strength.

Performance tests were conducted at room temperature (23 ± 0.5 °C). In order to test the tensile performance of the B-FWJ of the PE100 pipeline under accelerated hydrothermal subject to aging at various temperature levels, the specimens were processed and made into tensile specimens based on the GB/T 19810-2005 [27] and the corresponding international standard ISO 13953-2001 [28], and the tensile strength of the specimens was tested according to these specifications. This experiment used the ETM204C (Shenzhen Wance Equipment Co., Ltd., Shengzhen, China) microcomputer-controlled electronic universal testing machine to conduct tensile testing on the specimens. Figure 2 depicts a photo of the tensile specimen with a 50 mm/min stretching speed. The tensile strength of the samples was determined as the average value obtained from 5 parallel tests.

**Figure 3:**
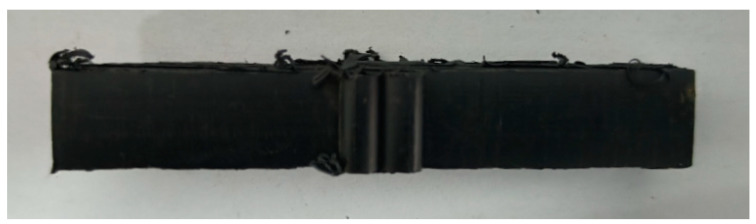
Photograph of a sample for mechanical impact testing.

In order to test the impact resistance of the B-FWJ of the PE100 pipeline under accelerated hydrothermal aging at various temperature levels, the specimens underwent processing and were prepared according to the Chinese national standard GB/T 1043.1-2008 [29] and the corresponding ISO 179-1-2023 [30]. The impact performance of the specimens was evaluated according to these specifications. In this experiment, the specimens’ impact resistance was tested using a PTM2200 pendulum testing machine (Shenzhen Sansi Zongheng Technology Co., Ltd., Shengzhen, China). Figure 3 displays a photo of the mechanical shock test sample. The parameters used for the impact test are outlined in Table 2, showing the impact strength testing of a simply supported beam. The impact energy and impact strength of the samples were calculated as the average values derived from 5 parallel tests.

**Figure 4:**
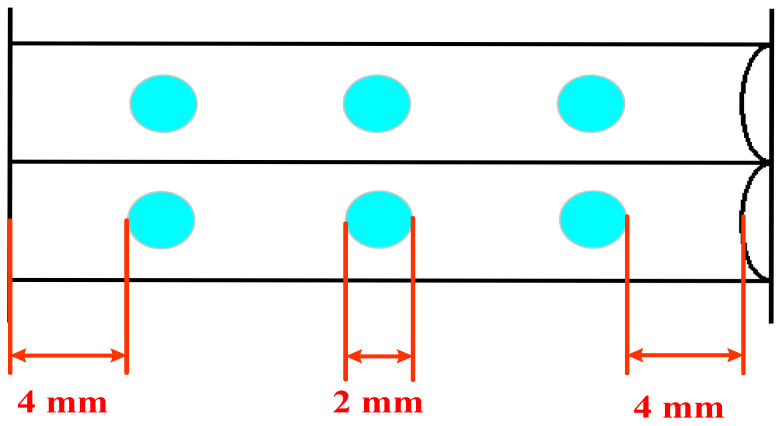
Schematic diagram showing the measurement points for hardness assessment.

The hardness test was conducted using the LX-D Shore hardness tester (Shenzhen yuanhengtong Technology Co., Ltd., Shengzhen, China), and the specimens were subjected to hardness testing following GB/T 38119-2019 [31]. Using the Shore hardness level, a total of 6 test points were selected on the surface of the B-FWJ specimen, depicted as blue circles in Figure 4. The average of the hardness values measured from five samples represents the Shore hardness of the sample.

Table 2 specifies the determination of the Vicat softening temperature (VST) and the preparation of specimens for B-FWJ [32]. The HVT302B microcomputer thermal control deformation Vicat softening point testing machine (Shenzhen Wance Testing Equipment Co., Ltd., Shengzhen, China) was used in this experiment. The sample’s VST was established as the mean result from three replicate experiments.

### 2.4. Characterization Technic

The OIT (oxidation induction time) was measured by DSC-500B differential scanning calorimetry. According to GB/T 19466.6-2009 [33] (corresponding to ISO 11357-6-2018 [34]), the thermal performance changes of PE100 pipeline B-FWJ specimens were assessed and characterized under different aging temperatures and conditions. The oxidation induction period of the sample was determined by taking the average of 3 parallel test outcomes.

Scanning electron microscopy (SEM) was used to observe the microstructure of the fractured surface after tensile fracture using Helios5 CX type SEM produced by FEI Company in the Hillsboro, OR, USA.

The FTIR-650S Fourier transform infrared spectrometer (Tianjin Gangdong Technology Co., Ltd., Tianjin, China) was used for infrared spectroscopy testing to describe the chemical composition of the sample’s exposed surface. The surface of the sample was carefully cleaned before conducting the testing. A wavenumber range of 400–6000 cm^−1^ was used. The signal-to-noise ratio was 45,000:1. The resolving power was 4 cm^−1^.

## 3. Results and Discussion

### 3.1. SEM

**Figure 5:**
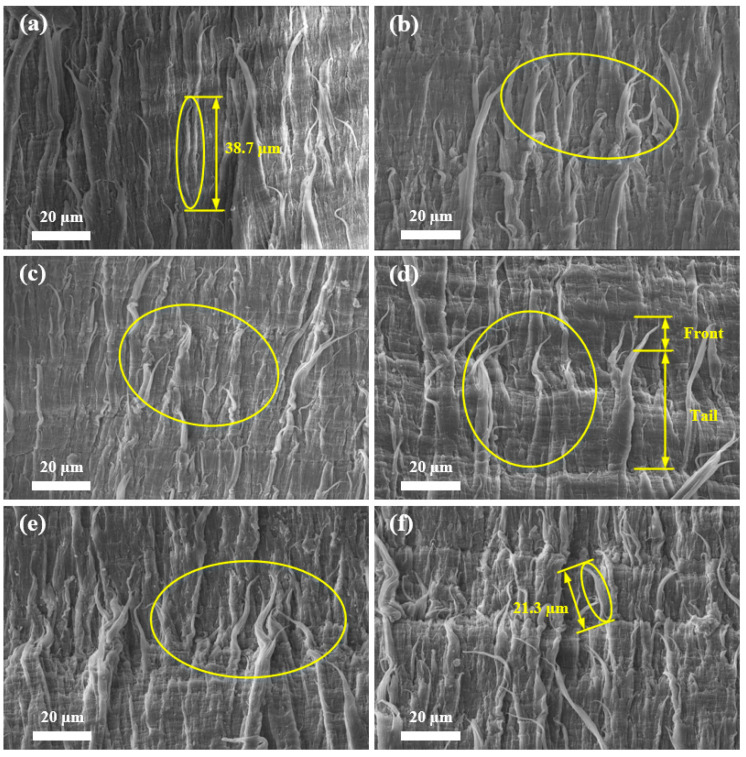
M-SM of the fracture surface resulting from the B-FWJ at five distinct time points without aging and hydrothermal aging at 40 °C: (**a**) not aged, (**b**) 120 h, (**c**) 240 h, (**d**) 360 h, (**e**) 480 h, and (**f**) 600 h. Yellow circles are used to highlight the parts where the fiber changes are more obvious.

Figure 5 shows the micro-surface morphology (M-SM) of the fracture surface resulting from the B-FWJ at five distinct time points without aging and hydrothermal aging at 40 °C. During the 600 h aging process, the fiber length of the B-FWJ decreased from 38.7 μm to 21.3 μm. At 0–480 h, the diameter and length of the fibers in the B-FWJ did not change significantly, but the number of fibers in the B-FWJ increased. Following 600 h of aging, the length of the fibers in the B-FWJ notably decreased, while the number of fibers reached its maximum.

**Figure 6:**
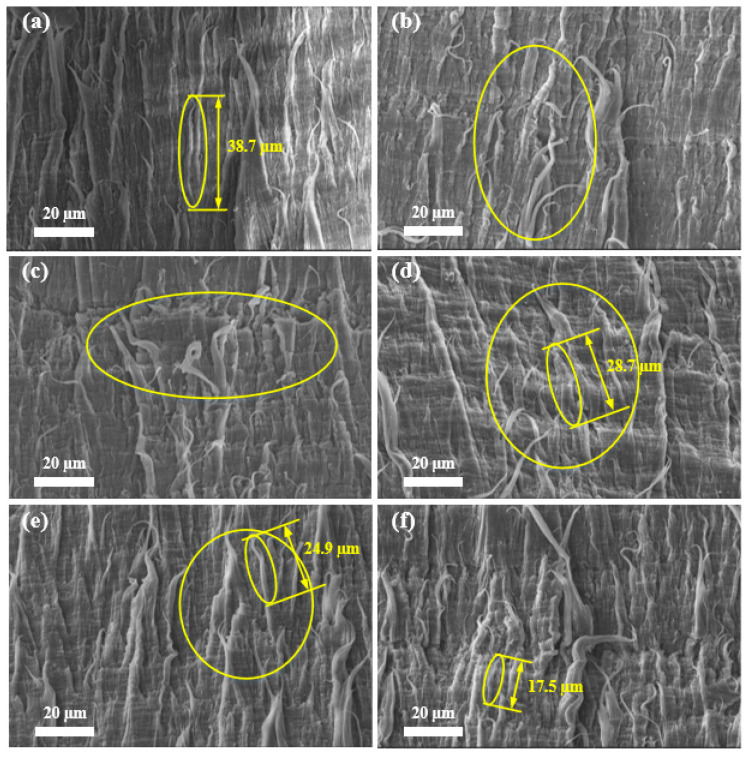
M-SM of the fracture surface resulting from the B-FWJ at five distinct time points without aging and hydrothermal aging at 60 °C: (**a**) not aged, (**b**) 120 h, (**c**) 240 h, (**d**) 360 h, (**e**) 480 h, and (**f**) 600 h. Yellow circles are used to highlight the parts where the fiber changes are more obvious.

Figure 6 reveals the M-SM of the fracture surface resulting from the B-FWJ at five distinct time points without aging and hydrothermal aging at 60 °C. The B-FWJ, which had undergone aging for less than 600 h, exhibited a significant reduction of 54.8% in fiber length. The aging time of 480 h can be considered the threshold for fiber changes. The fiber length of the B-FWJ aged 360 h was 3.8 μm shorter than that of the B-FWJ aged 480 h. The fiber length of a 600 h-aged B-FWJ was 7.4 μm longer than that of a 480 h-aged joint. Before the boundary, the diameter and length of the fibers in the B-FWJ did not change significantly, but the number of fibers in the B-FWJ showed an increasing trend. After the boundary, the length of the B-FWJ fibers significantly decreased, and the number of fibers was higher than that of the B-FWJ fibers before aging for 600 h.

**Figure 7:**
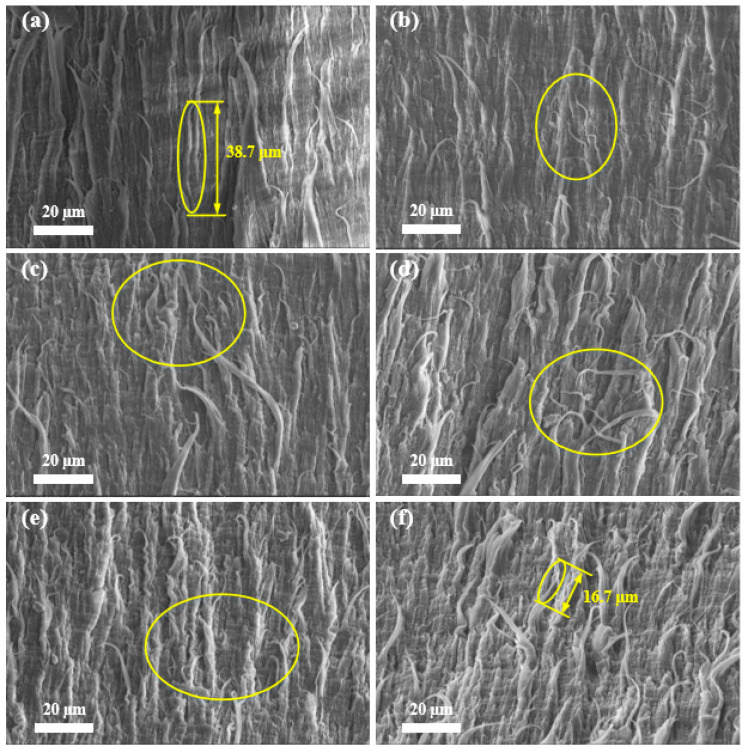
M-SM of the fracture surface resulting from the B-FWJ at five distinct time points without aging and hydrothermal aging at 80 °C: (**a**) not aged, (**b**) 120 h, (**c**) 240 h, (**d**) 360 h, (**e**) 480 h, and (**f**) 600 h. Yellow circles are used to highlight the parts where the fiber changes are more obvious.

Figure 7 displays the M-SM of the fracture surface resulting from the B-FWJ at five distinct time points, including before the aging and under the hydrothermal aging at 80 °C. After a duration of 600 h, the fiber length of the B-FWJ decreased significantly, from 38.7 to 17.5 μm—a total reduction of 56.8%. The hydrothermal aging in water at this temperature resulted in a shorter fiber length on the surface of the rupture of the B-FWJ compared with at 40 °C and 60 °C, respectively. The changes in the fractured surface fibers of B-FWJ aged under hydrothermal aging at different temperatures exhibited similarities. After 0–480 h, there was no significant change in the diameters and lengths of the fibers in the B-FWJ, but the number of fibers showed an increasing trend. After undergoing aging for 600 h, the length of the fibers in the B-FWJ underwent a notable decrease. And the number of fibers was higher than that of the B-FWJ fibers before aging for 600 h.

As the aging time and the aging temperature increased, the diameter and length of B-FWJ fibers did not change significantly in the first stage (0–120 h). However, in the second stage (120–480 h), the diameter and length of the fibers in the B-FWJ diminished. Following a 600 h aging process, the fractured surface of the B-FWJ exhibited the greatest roughness at 80 °C, characterized by numerous fiber breaks and a reduction in fiber length. The fractured surface of the B-FWJ was the roughest at 40 °C. Therefore, it can be concluded that the increase in temperature under hydrothermal aging conditions will accelerate the aging rate of the B-FWJ. When the specimen was subjected to axial tension, as the stretching continued, the molecular chains of the B-FWJ continued to be subjected to force, and the molecular chain morphology gradually tended towards a linear shape. Furthermore, as the distance between molecular chains gradually increased, the interaction force between molecular chains gradually decreased, and a reduction in the toughness of the B-FWJ was observed. The macroscopic manifestation was that the fractured surface produced fiber filaments and fiber filament fractures, and the toughness of the B-FWJ decreased.

As the duration of hydrothermal aging increased at a constant temperature, two phenomena were observed. Firstly, the antioxidant content of the B-FWJ gradually decreased, leading to aging and degradation of the material; secondly, the interaction between the material’s molecular chains and groups with water molecules led to changes in the structural characteristics of their interfaces [35]. Consequently, this reaction diminished the contribution of interaction forces between material molecular chains and molecular groups to the material’s strength, affirming the strength changes observed in Section 3.4 for the B-FWJ.

### 3.2. Oxidation Induction Time

DSC was utilized to measure the OIT of the B-FWJ following water bath aging at various temperatures. Previous research conducted by scholars has established the attenuation index equation for the OIT of polyethylene pipelines, represented by Equation (1), given below. The B-FWJ, a constituent of a polyethylene pipeline, followed the same attenuation equation for its OIT, as described below.

(1) $$T(t)=T_{0}\cdot e^{\left(-s\cdot t\right)}+A.$$

In Equation (1), *t* is the aging time of the B-FWJ, *T*(*t*) is the OIT of the B-FWJ after the aging time “*t*” in *h* hours, *T*_0_ is the OIT of the unprocessed B-FWJ, *s* is the antioxidant consumption rate, and A is a constant.

**Figure 8:**
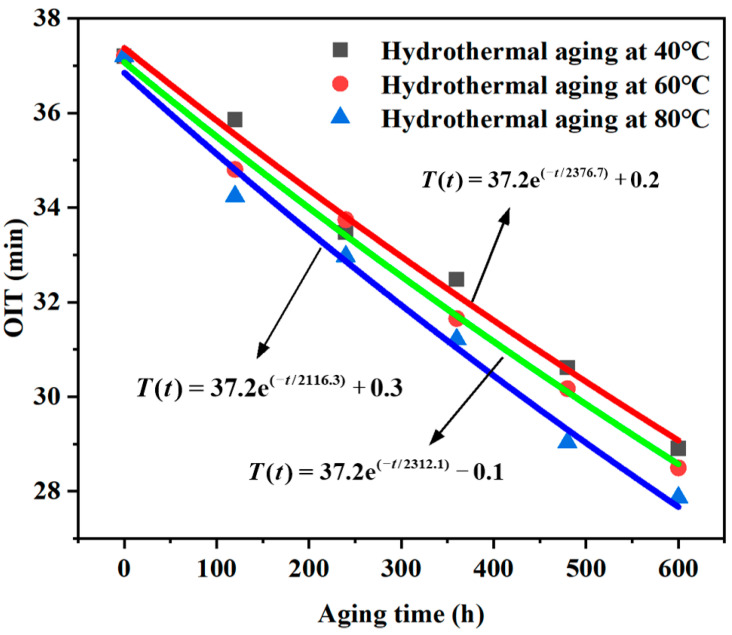
The fitting outcomes of the OIT decay index for the B-FWJ subsequent to thermal oxidative aging at varying temperatures.

Figure 8 depicts the fitting curve and corresponding equation, which characterize the decline in the OIT of the B-FWJ subjected to wet heat aging at differing temperatures. The DSC technique was employed to measure the OIT of the B-FWJ following hydrothermal aging at various temperatures. Figure 8 demonstrates that, as the aging duration increased, the antioxidants within the B-FWJ, which had undergone hydrothermal aging at various temperatures, underwent gradual consumption, resulting in a decreasing trend in their OIT values. The order of reduction in the oxidation induction period of B-FWJ subjected to hydrothermal aging at the same temperature, from highest to lowest, was as follows: 80 °C (25.00%), 60 °C (23.39%), and 40 °C (22.32%). The results are similar to the mechanical performance of the B-FWJ in Section 3.4. At the same aging time, as the temperature increased, the activity of antioxidants in the B-FWJ increased, leading to a higher rate of antioxidant consumption and a gradual decrease in the oxidation induction period of the B-FWJ. When the aging time was the same, the higher the temperature of hydrothermal aging, the lower the oxidation induction period value of the B-FWJ. At an identical aging time, an increase in the temperature of hydrothermal aging resulted in a decrease in the oxidation induction period value of the B-FWJ.

### 3.3. ATR FT-IR Spectroscopy

**Figure 9:**
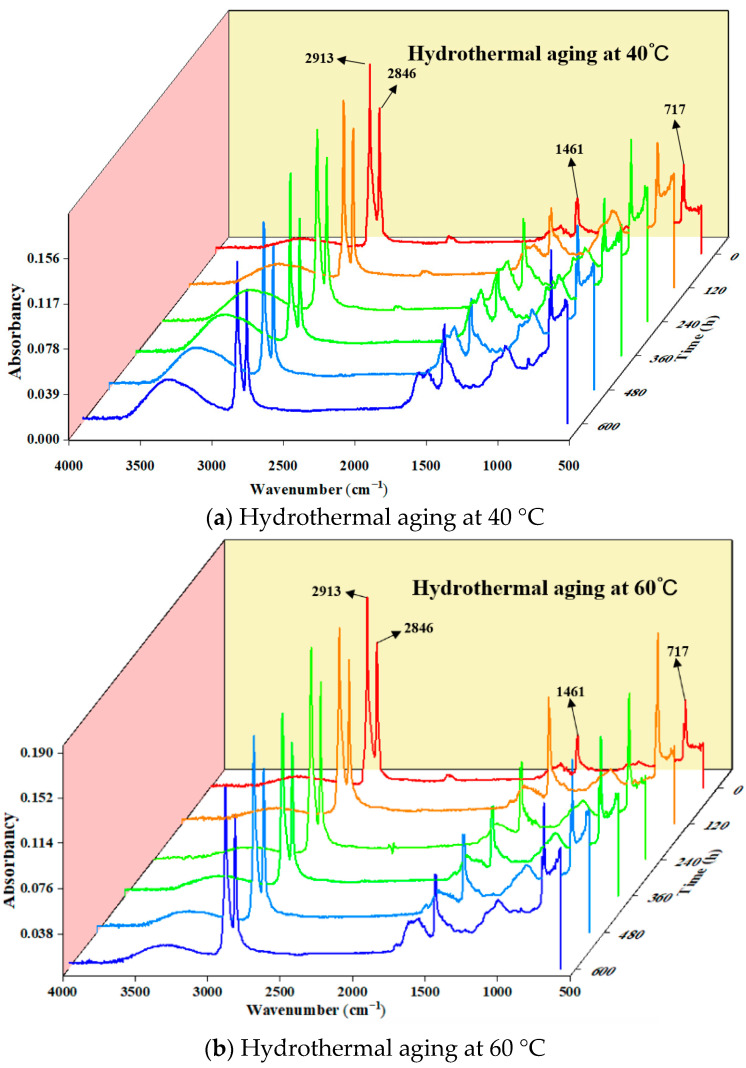

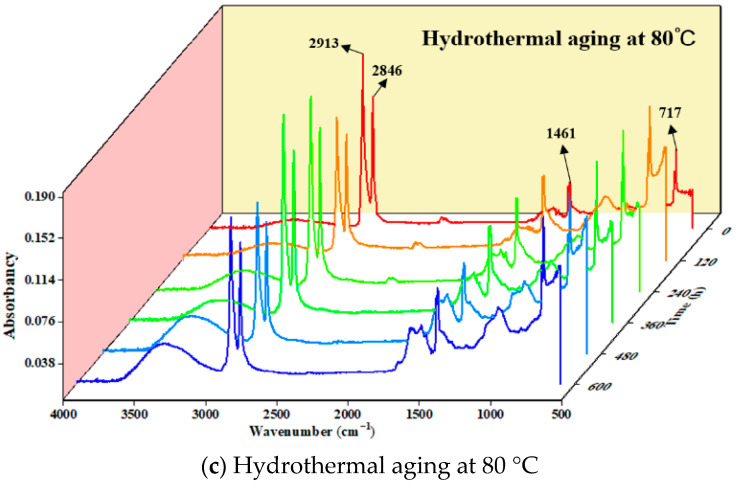
Absorbance waterfall diagram of B-FWJ with hydrothermal aging at various times.

To obtain the changes in the number of major functional groups during the aging procedure of HDPE B-FWJ, the commonly used method is to reflect the trend of the functional group changes through ATR testing. The asymmetric and symmetric tensile vibrations of methylene (-CH_2_) occur at 2913 cm^−1^ and 2846 cm^−1^, respectively. Additionally, the deformation of hydrocarbon bonds and the rocking vibrations of carbon–carbon single bonds occur at 1461 cm^−1^ and 717 cm^−1^, respectively. The infrared spectra of the unaged and aged B-FWJ samples were similar and had little change, so it can be considered that the carbon skeleton structure of the B-FWJ did not show significant changes in the hydrothermal aging accelerated aging test. After the aging of the B-FWJ of the pipeline, the molecular chain breakage in the microstructure increased [36]. By examining the infrared spectra of the B-FWJ sample in Figure 9, during the aging process at different temperatures, it became evident that hydrothermal aging did not cause significant damage to the basic material skeleton.

**Figure 10:**
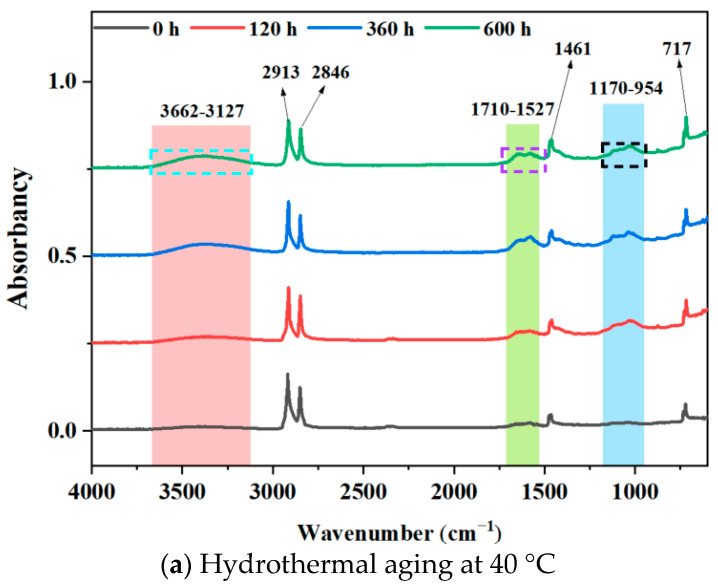

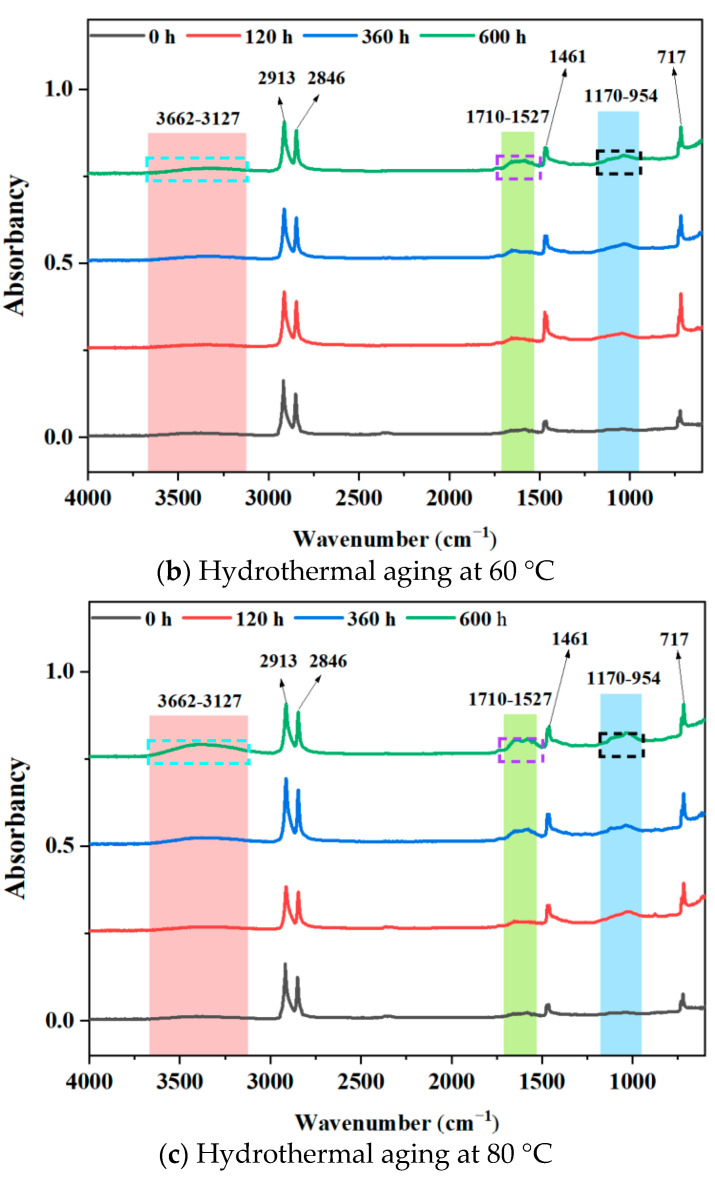
Absorbance spectra of B-FWJ aged by hydrothermal aging at various times.

As shown in Figure 10, the absorption peaks of the B-FWJ after the hydrothermal aging were in the ranges 3662–3127 cm^−1^, 1710–1527 cm^−1^, and 1170–954 cm^−1^. The functional groups within the 1710–1527 cm^−1^ band mainly consisted of carbonyl groups. The stretching vibrations of C-O bonds in carbonyl C=O, undamaged alcohol structures, and -CH_2_OH continuously increased with aging time within the 1710–1527 cm^−1^ and 1170–954 cm^−1^ bands [37]. The functional groups in the 1170–954 cm^−1^ band mainly consisted of ether groups, and a characteristic absorption peak of carbonyl groups appeared in this band [38]. The products of B-FWJ hydrothermal aging mainly included esters, ketones, aldehydes, and carboxylic acids, identified as polyethylene degradation products [39]. Furthermore, the presence of OH bonds could be attributed to a reaction between the water molecules and the B-FWJ molecular chain, and partly came from some molecular chains of B-FWJ and water molecules themselves [40]. Therefore, it is believed that, after aging the B-FWJ in this hydrothermal aging test, there were aging products such as alcohols, ethers, phenols, and hydrocarbons in the material.

**Figure 11:**
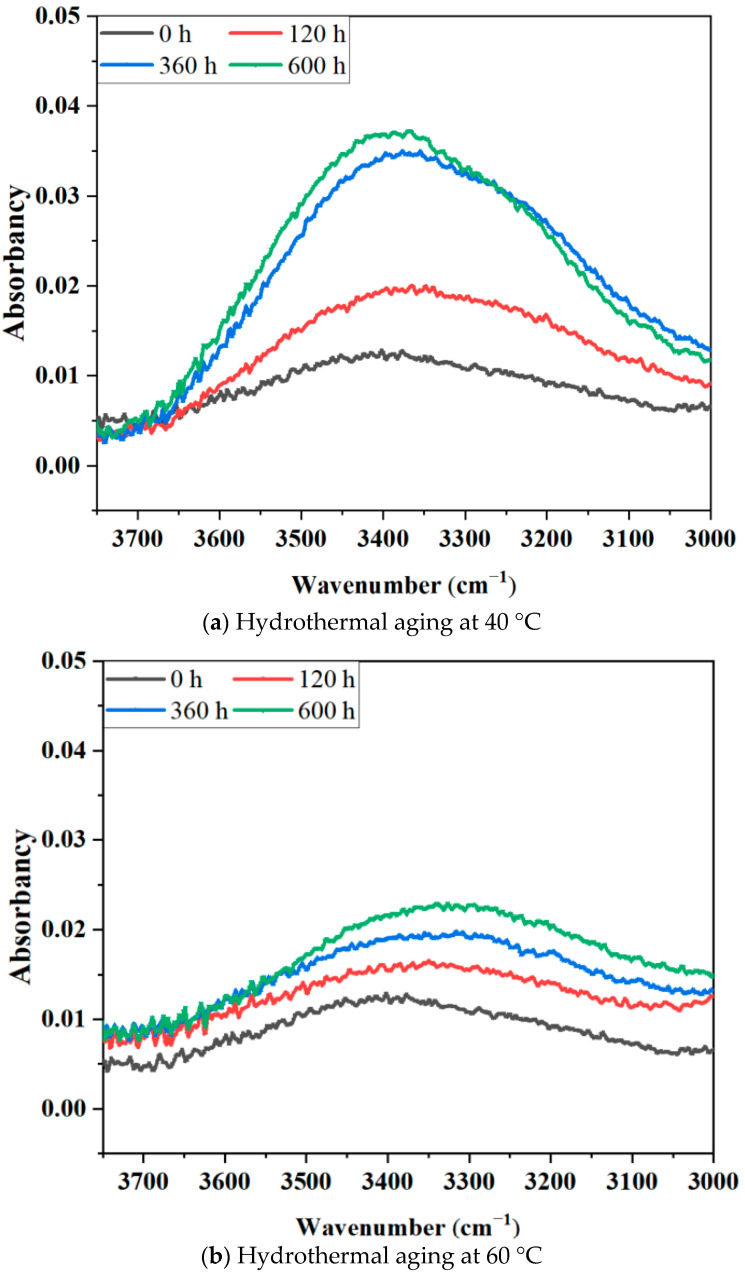

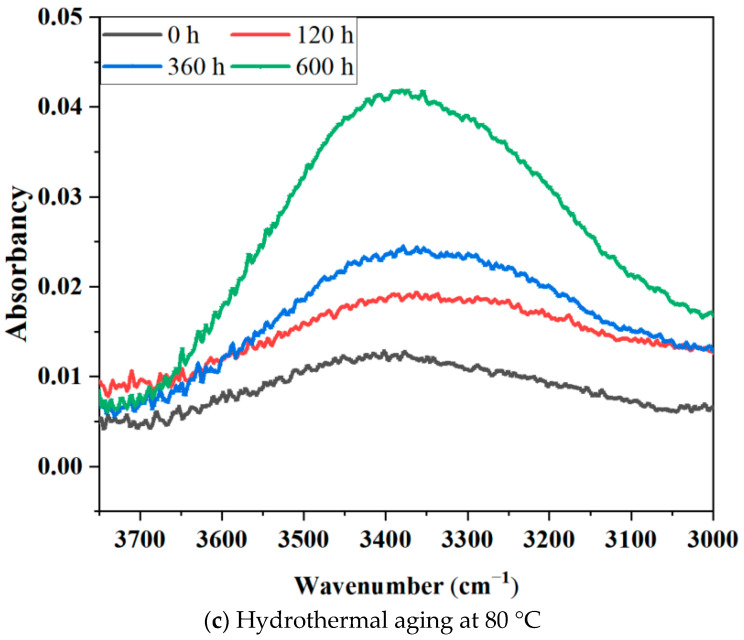
The absorbance spectra of B-FWJ aged by hydrothermal aging at various times, enlarged in the range of 3700–3000 (cm^−1^).

Figure 11 presents a magnified image of the B-FWJ within the 3662–3127 cm^−1^ wavelength range, following exposure to hydrothermal aging at various temperatures. At a constant hydrothermal aging temperature and within the same absorbance band, the absorbance peak of the B-FWJ increased as the aging duration extended. The characteristics of the sample in the 3662–3127 cm^−1^ band aligned with the vibrations of -OH and -C≡C-, providing further evidence for the formation of new substances or groups during the process of aging the B-FWJ.

### 3.4. Mechanical Properties

#### 3.4.1. Analysis of Impact Performance

**Figure 12:**
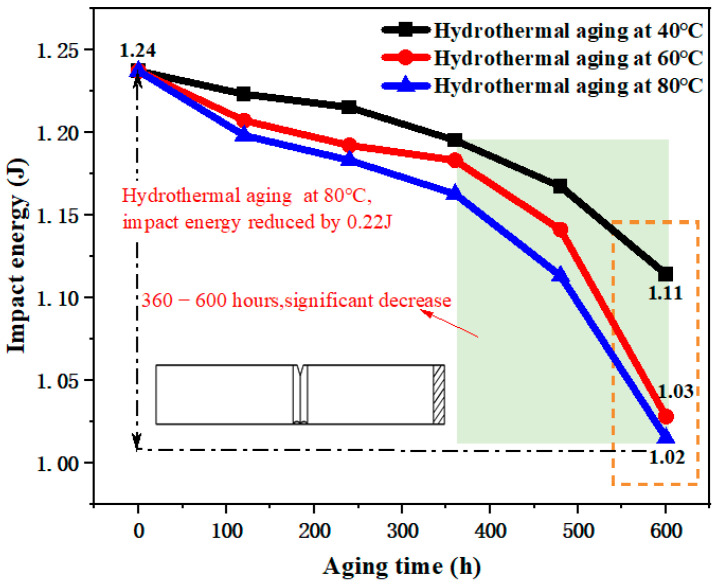
Impact energy of B-FWJ after hydrothermal aging at different temperatures.

As depicted in Figure 12, there was a negative correlation between the impact energy of the B-FWJ and the aging time after undergoing hydrothermal aging at various temperatures.

During the entire aging process, specifically in the aging period of 480–600 h at 80 °C in a water bath, the specimen’s B-FWJ impact energy decreased the most, reaching 0.09 J (a total decrease of 0.22 J), accounting for 40.91% of the total decrease. Moreover, the slope of the impact energy of the B-FWJ specimen was the largest. Figure 12 shows the impact energy variation curve of the thermal butt-fusion joint following hydrothermal aging at varying temperatures. The impact energy change of the B-FWJ specimen during the aging process, particularly in the hydrothermal aging at 60 °C, was similar to the impact energy change of the B-FWJ specimen at 80 °C during hydrothermal aging. When the aging time reached 600 h, the decrease in impact energy (0.13 J reduction) of the B-FWJ after hydrothermal aging at 40 °C was comparatively smaller than the reductions observed in the B-FWJ after hydrothermal aging at 80 °C and 60 °C (0.22 J reduction for hydrothermal aging at 80 °C and 0.21 J reduction for hydrothermal aging at 60 °C).

**Figure 13:**
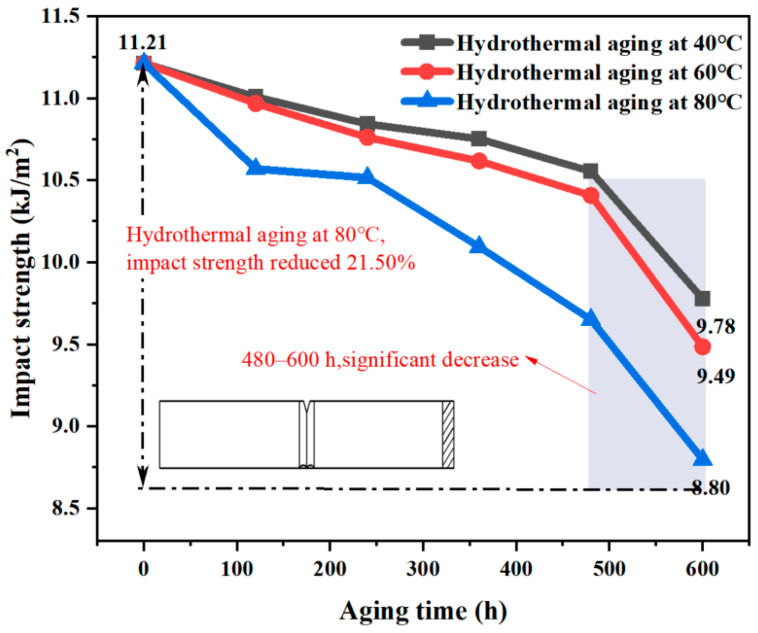
The B-FWJ’s impact strength diminishes as it undergoes hydrothermal aging at varying temperatures.

As illustrated in Figure 13, the B-FWJ’s impact strength diminished as it underwent hydrothermal aging at varying temperatures. As the aging duration increased, the impact strength diminished. Notably, after the aging process, the B-FWJ subjected to hydrothermal aging at 80 °C exhibited a significant reduction in impact strength, decreasing by 21.50% compared with the other two temperature conditions. Conversely, the B-FWJ showed the least decrease in impact strength when aged at 60 °C, with a mere reduction of 12.76%. When the aging time was the same, an increase in the test temperature made long-chain breakage in the molecules within the hydrothermal environment easier. This resulted in an increased number of short chains, a reduced difficulty in intermolecular cross-linking, increased brittleness of the material, and a subsequent decrease in both the impact energy and impact strength of the sample. Therefore, the impact energy and strength of B-FWJ were highly affected by temperature under the hydrothermal aging conditions.

#### 3.4.2. Tensile Properties

**Figure 14:**
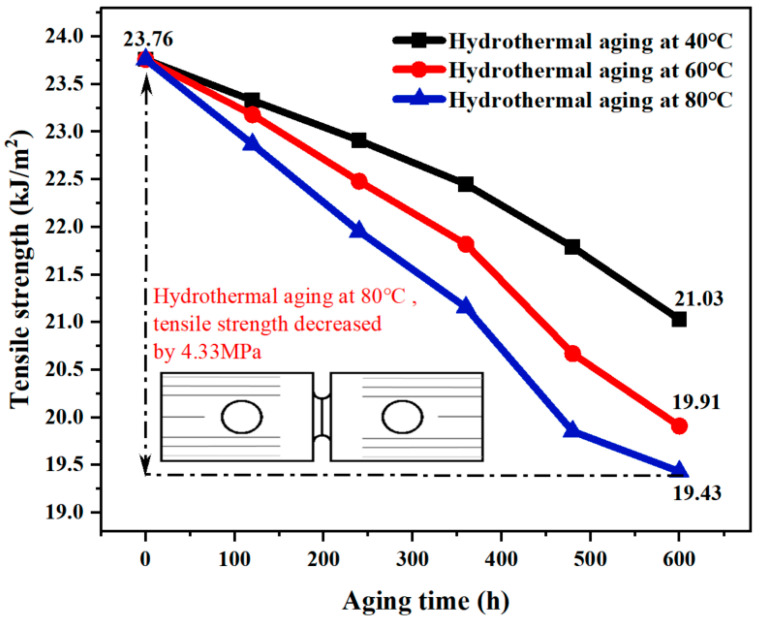
The B-FWJ’s tensile strength diminishes as it undergoes hydrothermal aging at varying temperatures.

Figure 14 shows that the B-FWJ’s tensile strength diminished as it underwent hydrothermal aging at varying temperatures There was a negative correlation between the tensile strength of the B-FWJ, aging time, and temperature. After 600 h of aging, the tensile strength of the B-FWJ, regardless of the aging temperature, demonstrated comparable values. Following the completion of the aging process, the tensile strength of the B-FWJ exhibited a reduction of 4.33 MPa after hydrothermal aging at 80 °C and a decrease of 2.73 MPa after hydrothermal aging at 40 °C.

As the aging time increased, the molecular bonds in the molecular chain of the B-FWJ became more susceptible to fracture or breakage, reducing the interaction force between the molecular chains. Hence, as the aging duration extended, the tensile strength of the B-FWJ diminished. When the aging time remained constant, increasing the temperature resulted in higher energy absorption by certain molecular chains in the B-FWJ (without reaching the energy required for disconnecting the bonds), reducing the molecular chains’ ability to resist tensile damage. Some molecular chains within the B-FWJ may also experience breakage. Under the combined action of molecular bond energy absorption and molecular chain breakage, the flexibility of the material was reduced. Due to the combined effects of molecular bond energy absorption and molecular chain rupture, the material’s flexibility underwent a reduction.

#### 3.4.3. Hardness

**Figure 15:**
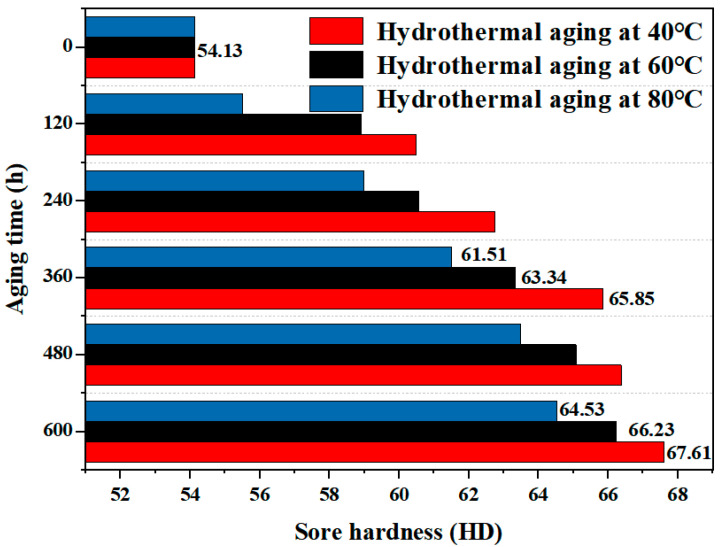
The B-FWJ’s surface hardness as it undergoes hydrothermal aging at varying temperatures.

Figure 15 shows the B-FWJ’s surface hardness as it underwent hydrothermal aging at varying temperatures. After conducting hydrothermal aging tests at three distinct temperatures, the surface hardness of the B-FWJ samples increased. Notably, after 600 h of aging, the sample aged at 80 °C exhibited the greatest increase in surface hardness, amounting to 24.90%. The increase in surface hardness of the B-FWJB-FWJ was the smallest at 40 °C during hydrothermal aging, reaching 19.21%. As time went on, the hardness of the B-FWJ increased in a stepwise manner after the hydrothermal aging at different temperatures. The degree of stepwise growth in surface hardness during the pre-aging stage (0–360 h) was more significant compared with the post-aging stage (360–600 h), indicating a gradual stabilization of material aging over time.

Since the PE pipe B-FWJ is a product formed through secondary processing, the crystallization zone of the B-FWJ is mainly divided into two types: the semi-crystalline zone and the crystalline zone. The molecular chains within the semi-crystalline region remain intact, having not yet experienced degradation or oxidation reactions with oxides.

The molecular chain has a low degree of crystallinity, and its structure maintains a high porosity, providing a loose flow gap for water and oxygen molecules to flow between the polymeric chains in the semi-crystalline area [41]. At the same aging time, as the hydrothermal aging temperature increases, the activity of oxygen and water molecules increases, and the number of them diffusing to the semi-crystalline region increases. The molecular chains undergo oxidation during the first stage and generate short chains. In the second stage, there is a shift in the arrangement of the molecular chains within the semi-crystalline region, resulting in a decrease in molecular chain spacing and a more orderly arrangement. Microscopic manifestation is that the crystallization zone of the B-FWJ expands, and its crystallinity increases. On a macroscopic level, the toughness of the B-FWJ decreases, the plasticity increases, and the hardness increases [42]. Gulmine et al. [43] conducted accelerated aging tests on polyethylene cables and found that the aging process increases the hardness and molecular chains of the polyethylene material.

#### 3.4.4. Vicat Softening Temperature

**Figure 16:**
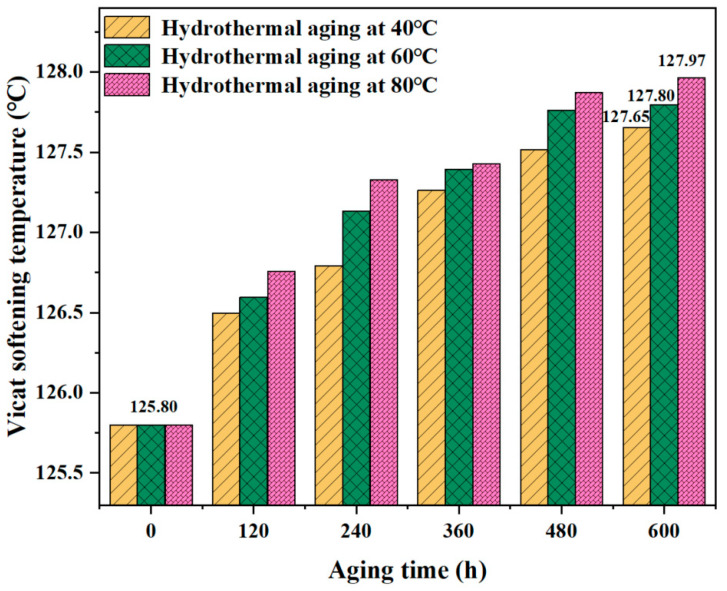
The B-FWJ’s VST as it undergoes hydrothermal aging at varying temperatures.

Figure 16 shows the B-FWJ’s VST as it underwent hydrothermal aging at varying temperatures. During the 0–480 h period, the VST of the B-FWJ showed an increasing trend over time. At an aging time of 600 h, the VST of the B-FWJ peaked after hydrothermal aging at three different temperatures. Still, the Vicat temperature change during the aging was less than 1.8%, indicating that hydrothermal aging has an insignificant effect on the VST of the B-FWJ.

As the hydrothermal aging temperature rose at the same aging time, the B-FWJ experienced a higher degree of molecular chain cross-linking and entanglement, resulting in alterations in the relative positions of molecules and molecular chains, ultimately leading to a decrease in the mobility of the molecular chains.

### 3.5. Discussion on Ageing Mechanisms

As the B-FWJv is the product of secondary processing, part of the molecular chain of the B-FWJ in the heat-affected zone is destroyed during the heating process. It results in a molecular chain break and release of hydroxyl and carbonyl groups that affect the mechanical properties of materials. During the melting process, the molecular chains of the B-FWJ undergo cross-linking and fracture, producing free hydroxyl and carbonyl groups. A portion of the long chains of molecules forms a grid-like structure due to cross-linking. Some of the long chains of molecules undergo breakage, shortening their length and producing molecular chain breaks and free groups, such as hydroxyl and carbonyl groups.

**Figure 17:**
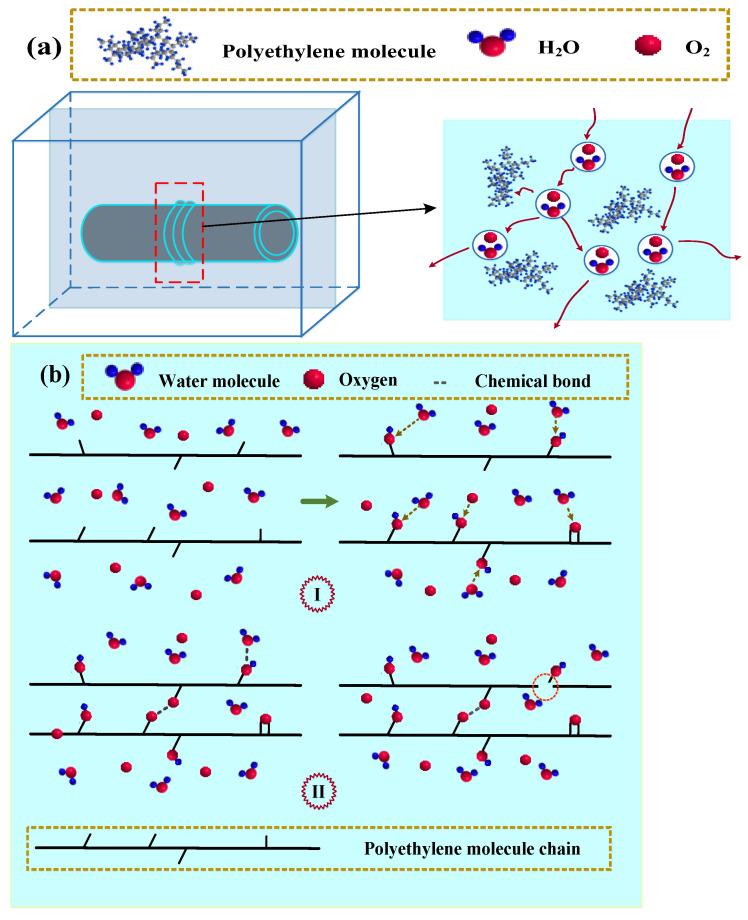
The process of water molecule infiltration on the surface of the B-FWJ and the mechanism of aging resulting from the interaction between the B-FWJ and water molecules. (**a**) Schematic diagram of the flow of water molecules. (**b**) The mechanism of aging resulting from the interaction between the B-FWJ and water molecules.

Figure 17 illustrates the process of water molecule infiltration on the surface of the B-FWJ and the mechanism of aging resulting from the interaction between the B-FWJ and water molecules. With an increase in aging time, there was a corresponding increase in the number of water molecules that permeated into the B-FWJ. The red framed area in Figure 17a depicts the B-FWJ, and the schematic diagram on its right side illustrates the penetration of water molecules into the B-FWJ. As the aging temperature rose and the degree of water molecule diffusion into the B-FWJ intensified, leading to the gradual consumption of antioxidants. The molecular chains of the oxidized B-FWJ underwent cross-linking and fracture. Due to the combined effect of temperature and water molecules, the aging reaction of the B-FWJ was severe in the early stage of hydrothermal aging. The reaction between the water molecules, oxygen, and the molecular chains of the B-FWJ led to the formation of alcohols, phenols, aldehydes, and ketones at the ends of the primary and secondary chains, indicating the initiation of aging in the B-FWJ, as shown in Phase I of Figure 17b. The macroscopic manifestation was the reduction in the material’s tensile strength and impact strength. Adjacent molecular chains underwent cross-linking reactions, producing -COOR (esters, carboxylic acids), increasing their molecular weight and reducing their flowability, as shown in Phase II of Figure 17b. This structural change made it more difficult for the material to undergo deformation when subjected to external forces, resulting in higher hardness and VST. As the reaction between the molecular chains of the B-FWJ, water molecules, and oxygen progressed, the degree of cross-linking and fracture in the long chains of the B-FWJ molecules increased, exacerbating the aging process. Aging exerted a notable influence on the mechanical properties and hardness of B-FWJ. On the one hand, aging caused a decline in the material’s tensile strength and impact strength. On the other hand, due to cross-linking reactions and structural alterations in the molecular chains, the material’s hardness and VST increased. Consequently, when assessing the impact of aging on B-FWJ, it is imperative to take into account its dual effects on both mechanical properties and either hardness or VST.

**Figure 18:**
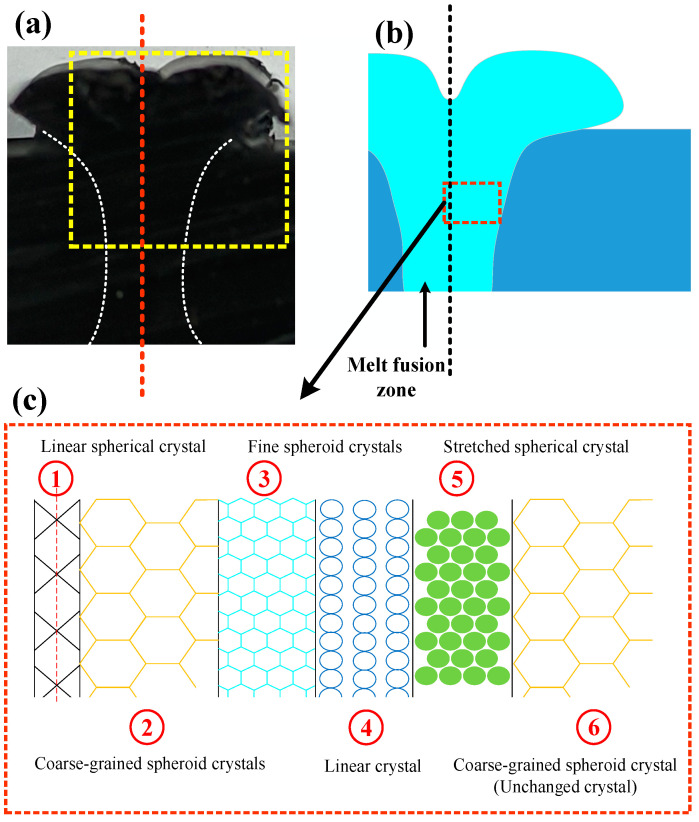
Schematic diagram of the crystallization zone around the weld seam of the B-FWJ: (**a**) partial physical diagram of the joint, (**b**) partial schematic diagram of the joint, (**c**) distribution diagram of the crystallization area on the right side of the joint weld.

The schematic diagram of the crystallization zone around the B-FWJ weld seam is shown in Figure 18. In Figure 18a, the area within the yellow framed box is selected to create a schematic diagram of the B-FWJ, which is shown in Figure 18b. In this schematic, the red line in Figure 18a and the dashed black line in Figure 18b represent the centerline of the B-FWJ. Figure 18c illustrates the division of the centerline of the B-FWJ into six layers within the red dashed box in (b), based on the different crystal structures along the weld seam towards the unmelted region. The centerline area of the B-FWJ weld seam is composed of linear spherical crystals. The area of the centerline of the B-FWJ weld seam was composed of linear spherical crystals. Spherical coarse-grained crystals were formed in the parts with low heat dissipation rates. Due to the increase in the heat dissipation rate from the inside out, the crystal morphology gradually changed into a spherical fine-grained crystalline layer from the part with a low heat dissipation rate to the part with a high heat dissipation rate. The melt region was composed of directional linear crystallization, and the interaction between the two parts of the tube during the melting welding process and the cooling of the joint produce directional linear crystallization. A portion of stretched spherical crystals was generated in the secondary and third layers, with a morphology similar to directional linear crystallization in the melt region. The stretched spherical crystal structure in the fifth layer was connected to the spherical coarse-grained crystal in the outermost unmelted region.

## 4. Conclusions

The accelerated aging tests performed on PE100 pipeline B-FWJ involved three hydrothermal aging cycles with a duration of 600 h. The results demonstrated that the impact resistance, tensile strength, and chemical properties of PE100 pipeline B-FWJ decreased with increasing aging time and temperature. Conversely, the VST and Shore hardness exhibited slight increases. These changes can be attributed to the enhanced reaction between the molecular chains of the B-FWJ and water molecules, accompanied by a broader range of cross-linking and molecular chain fracture. Notably, as the aging temperature rose, the alterations in the number and morphology of fiber filaments on the fractured surface of the B-FWJ could be divided into two stages. In the initial stage, no significant changes occurred in the number and morphology of fiber filaments. However, in the subsequent stage, the number of fiber strands sharply increased, while their length and width decreased. Additionally, the roughness of the fractured surface of the B-FWJ significantly increased due to these changes.

During the initial phase of hydrothermal aging, the B-FWJ underwent a pronounced aging reaction due to the combined influence of temperature and water molecules. This reaction involved the interaction between water molecules, oxygen, and the molecular chains of the welded joint, resulting in the formation of alcohols, phenols, aldehydes, and ketones at the terminals of both primary and secondary chains. This marked the onset of aging in the B-FWJ. Adjacent molecular chains underwent cross-linking reactions, This process increased the molecular weight of the molecular chains and decreased its flowability. Aging exerted a notable influence on the mechanical properties and hardness of B-FWJ. On the one hand, aging caused a decline in the material’s tensile strength and impact strength. On the other hand, due to cross-linking reactions and structural alterations in the molecular chains, the material’s hardness and VST increased. Consequently, when assessing the impact of aging on B-FWJ, it is imperative to take into account its dual effects on both mechanical properties and either hardness or VST.

## Figures and Tables

**Table 1:** Accelerated aging scheme.

Specimen	Accelerated Aging Type	Temperature	Aging Conditions
1	New pipeline	0 °C	—
2	Hydrothermal aging	40 °C	Hydrothermal aging
3		60 °C	
4		80 °C	

**Table 2:** Mechanical shock test parameters.

Impact Velocity (m/s)	Pendulum Energy (J)	Room Temperature (°C)
2.9	4	23 ± 0.5

## Data Availability

The original contributions presented in the study are included in the article, further inquiries can be directed to the corresponding author.

## References

[1] Desai U., Sharma B.K., Singh A., Singh A. (2020). Enhancement of resistance against damp heat aging through compositional change in PV encapsulant poly (ethylene-co-vinyl acetate). Sol. Energy.

[2] Hsueh H.C., Kim J.H., Orski S., Fairbrother A., Jacobs D., Perry L., Sung L. (2020). Micro and macroscopic mechanical behaviors of high-density polyethylene under UV irradiation and temperature. Polym. Degrad. Stab..

[3] Schulte U. A vision becomes true: 50 years of pipes made from High Density Polyethylene. Proceedings of the of Plastic Pipes XIII.

[4] Frank A., Pinter G., Lang R.W. (2009). Prediction of the remaining lifetime of polyethylene pipes after up to 30 years in use. Polym. Test..

[5] Shi X.M., Zhang J., Li D.R. (2009). Effect of damp-heat aging on the structures and properties of ethylene-vinyl acetate copolymers with different vinyl acetate contents. J. Appl. Polym. Sci..

[6] Agrawal P., Silva M.H., Cavalcanti S.N., Freitas D.M., Araújo J.P., Oliveira A.D., Mélo T.J. (2022). Rheological properties of high-density polyethylene/linear low-density polyethylene and high-density polyethylene/low-density polyethylene blends. Polym. Bull..

[7] Zha S., Lan H., Huang H. (2022). Review on lifetime predictions of polyethylene pipes: Limitations and trends. Int. J. Press. Vessel. Pip..

[8] Khademi-Zahedi R. (2019). Application of the finite element method for evaluating the stress distribution in buried damaged polyethylene gas pipes. Undergr. Space.

[9] Yang X.L., Wang S.H., Gong Y., Yang Z.G. (2021). Effect of biological degradation by termites on the abnormal leakage of buried HDPE pipes. Eng. Fail. Anal..

[10] Shete M.T., Yarasu R.B. (2021). Experimental investigation and finite element simulation of friction stir spot welding (FSSW) of high-density polyethylene joints. Mater. Today: Proc..

[11] Zheng J., Zhong S., Shi J., Guo W. (2015). Study on the Allowable Temperature for Preventing over Welding During Thermal Welding of Polyethylene Pipe. J. Press. Vessel. Technol..

[12] Vijayan V., Pokharel P., Kang M.K., Choi S. (2016). Thermal and mechanical properties of e-beam irradiated butt-fusion joint in high-density polyethylene pipes. Radiat. Phys. Chem..

[13] Nguyen K.Q., Mwiseneza C., Mohamed K., Cousin P., Robert M., Benmokrane B. (2021). Long-term testing methods for HDPE pipe-advantages and disadvantages: A review. Eng. Fract. Mech..

[14] Grabmayer K., Wallner G.M., Beißmann S., Braun U., Steffen R., Nitsche D., Lang R.W. (2014). Accelerated aging of polyethylene materials at high oxygen pressure characterized by photoluminescence spectroscopy and established aging characterization methods. Polym. Degrad. Stab..

[15] Kim C. (2019). Investigation on dielectric breakdown behavior of thermally aged cross-linked polyethylene cable insulation. Polym. Test..

[16] Chen G., Yang Y., Zhou C., Zhou Z., Yan D. (2019). Thermal-oxidative aging performance and life prediction of polyethylene pipe under cyclic and constant internal pressureThermal-oxidative aging performance and life prediction of polyethylene pipe under cyclic and constant internal pressure. J. Appl. Polym. Sci..

[17] Weon J.I. (2010). Effects of thermal ageing on mechanical and thermal behaviors of linear low density polyethylene pipe. Polym. Degrad. Stab..

[18] Chen H., Scavuzzo R.J., Srivatsan T. (1997). SInfluence of joining on the fatigue and fracture behavior of high density polyethylene pipe. J. Mater. Eng. Perform..

[19] Bowman J. (1997). A review of the electrofusion joining process for polyethylene pipe systems. Polym. Eng. Sci..

[20] Chebbo Z., Vincent M., Boujlal A., Gueugnaut D., Tillier Y. (2015). Numerical and experimental study of the electrofusion welding process of polyethylene pipes. Polym. Eng. Sci..

[21] Stacer R.G., Schreuder-Stacer H.L. (1989). Time-dependent autohesion. Structural Integrity: Theory and Experiment.

[22] Lee P.A., Kim S., Stakenborghs B., Suh Y., Choi S. (2022). Development of hydro-axial tension method for whole pipe butt-fusion joint tensile test. Polym. Test..

[23] Wermelinger J., Hein O. Long term influence of defects in butt fusion joints on PE 100 pipes and NDT detection of them. Proceedings of the Plastic Pipes Conference PPXVIII.

[24] Kim J.S., Oh Y.J., Choi S.W., Jang C. (2019). Investigation on the thermal butt fusion performance of the buried high density polyethylene piping in nuclear power plant. Nucl. Eng. Technol..

[25] Ghanadi M., Padhye L.P. (2024). Revealing the long-term impact of photodegradation and fragmentation on HDPE in the marine environment: Origins of microplastics and dissolved organics. J. Hazard. Mater..

[26] Liu Y., Akula K.C., Dandamudi K.P.R., Liu Y., Xu M., Sanchez A., Zhu D., Deng S. (2022). Effective depolymerization of polyethylene plastic wastes under hydrothermal and solvothermal liquefaction conditions. Chem. Eng. J..

[27] (2005). Polyethylene(PE)pipe and Fittings-Determination of the Tensile Strength and Failure Mode of Test Pieces from a Butt-Fused Joint.

[28] (2001). Polyethylene (PE) Pipes and Fittings—Determination of the Tensile Strength and Failure Mode of Test Pieces from a Butt-Fused Joint.

[29] (2008). Plastics—Determination of Charpy Impact Properties—Part 1: Non-Instrumented Impact Test.

[30] (2023). Plastics—Determination of Charpy Impact Properties—Part 1: Non-Instrumented Impact Test.

[31] (2019). Verification of Shore Durometers.

[32] (1995). Thermoplastics Pipes and Fittings—Vicat Softening Temperature—Part 1: General Test Method.

[33] (2009). Plastics—Differential Scanning Calorimetry (DSC)—Part 6: Determination of Oxidation Induction Time (Isothermal OIT) and Oxidation Induction Temperature (Dynamic OIT).

[34] (2018). Plastics. Differential Scanning Calorimetry (DSC). Part 6: Determination of Oxidation Induction Time (Isothermal OIT) and Oxidation Induction Temperature (Dynamic OIT).

[35] Kovács R.L., Csontos M., Gyöngyösi S., Elek J., Parditka B., Deák G., Erdélyi Z. (2021). Surface characterization of plasma-modified low density polyethylene by attenuated total reflectance fourier-transform infrared (ATR-FTIR) spectroscopy combined with chemometrics. Polym. Test..

[36] Kemari Y., Mekhaldi A., Teyssèdre G., Teguar M. (2019). Correlations between structural changes and dielectric behavior of thermally aged XLPE. IEEE Trans. Dielectr. Electr. Insul..

[37] Allen N.S., Palmer S.J., Marshall G.P., Luc-Gardette J. (1997). Environmental oxidation processes in yellow gas pipe: Implications for electrowelding. Polym. Degrad. Stab..

[38] Maria R., Rode K., Schuster T., Geertz G., Malz F., Sanoria A., Brendlé E. (2015). Ageing study of different types of long-term pressure tested PE pipes by IR-microscopy. Polymer.

[39] Montes J.C., Cadoux D., Creus J., Touzain S., Gaudichet-Maurin E., Correc O. (2012). Ageing of polyethylene at raised temperature in contact with chlorinated sanitary hot water. Part I–Chemical aspects. Polym. Degrad. Stab..

[40] Rozental-Evesque M., Martin F., Bourgine F., Colin X., Audouin L., Verdu J. (2006). Etude du Comportement de Tuyaux en Polyethylene Utilises Pour le Transport D’eau Potable en Presence de Desinfectant Chlore.

[41] Gong Y., Wang S.H., Zhang Z.Y., Yang X.L., Yang Z.G., Yang H.G. (2021). Degradation of sunlight exposure on the high-density polyethylene (HDPE) pipes for transportation of natural gases. Polym. Degrad. Stab..

[42] Dörner G., Lang R.W. (1998). Influence of various stabilizer systems on the ageing behavior of PE–MD—II. Ageing of pipe specimens in air and water at elevated temperatures. Polym. Degrad. Stab..

[43] Gulmine J.V., Janissek P.R., Heise H.M., Akcelrud L. (2003). Degradation profile of polyethylene after artificial accelerated weathering. Polym. Degrad. Stab..

